# Comment on: Managing sickle cell disease and related complications in pregnancy: results of an international Delphi panel

**DOI:** 10.1186/s12884-026-09721-x

**Published:** 2026-07-29

**Authors:** Deva Sharma, Ilknur Pamuk, Kenneth I. Ataga, Alexandra Benachi, Selim Büyükkurt, Sophie Lanzkron, Hakan Ozdogu, Shivan Pancham, Lydia H. Pecker, Susan E. Robinson, Safak Yilmaz Baran

**Affiliations:** 1 Departments of Medicine and Pathology, Microbiology and Immunology, Divisions of Hematology-Oncology & Transfusion Medicine, Vanderbilt University Medical Center, Nashville, TN USA; 2 Department of Physiology, Faculty of Medicine, Başkent University, Ankara, Turkey; 3 Adana Adult Bone Marrow Transplantation Center (CIC. 589), Apheresis Unit, Başkent University, Adana, Yuregir Turkey; 4 Center for Sickle Cell Disease, University of Tennessee Health Science Center, Memphis, TN USA; 5 Department of Obstetrics and Gynecology, “Antoine Béclère” Hospital, Assistance Publique–Hôpitaux de Paris, Paris Saclay University Hospitals, Clamart, France; 6 Department of Obstetrics and Gynecology, Faculty of Medicine, Çukurova University, Adana, Turkey; 7 Department of Medicine, Division of Hematology, Johns Hopkins School of Medicine, Baltimore, MD USA; 8 Department of Hematology, Faculty of Medicine, Başkent University, Ankara, Turkey; 9 Department of Haematology, Sandwell and West Birmingham Hospitals National Health Service Trust, West Bromwich, UK; 10 Department of Gynecology and Obstetrics, Johns Hopkins University School of Medicine, Baltimore, MD USA; 11 Department of Women’s Health, Guy’s and St Thomas’ National Health Service Foundation Trust, London, UK; 12 Department of Obstetrics and Gynecology, Başkent University, Dr. Turgut Noyan Application and Research Center, Adana, Turkey; 13 Department of Medicine, Division of Hematology, Thomas Jefferson University, Philadelphia, PA USA

**Keywords:** Sickle cell disease, High-risk pregnancy, Antenatal care, Vaso-occlusion, Prenatal counseling, Anemia, Maternal complications, Fetal outcomes, Red blood cell transfusions, Knowledge gaps

## Abstract

Consensus-based recommendations on managing sickle cell disease in pregnancy were recently published in a hematology journal. As this topic is also of great interest to obstetricians and gynecologists, we summarize some of these recommendations, while highlighting the challenges of providing evidence-based medical care to pregnant individuals with sickle cell disease.

## Background

Sickle cell disease (SCD) is a genetic blood disorder characterized by the presence of sickle hemoglobin (HbS), a variant of normal adult hemoglobin. When deoxygenated, HbS polymerizes and damages the red blood cells that contain it, which become crescent- or sickle-shaped. These red blood cells are prone to hemolysis and contribute to vaso-occlusion and ischemia. Individuals with SCD can experience a wide range of acute and chronic complications with variable severity, including but not limited to anemia, vaso-occlusive pain episodes, acute chest syndrome (an acute illness characterized by fever and/or respiratory symptoms and new pulmonary infiltrate on chest X-ray), venous thromboembolism, and stroke [1].

Owing to advances made in early diagnosis and treatment (including disease-modifying therapies like hydroxyurea and red blood cell transfusions), the life expectancy of individuals with SCD has improved significantly over the past decade in high-income countries, where SCD is now considered a chronic disease [1]. Nevertheless, SCD represents a large and growing public health concern, as in 2021, over 7.7 million people were estimated to be living with SCD globally, and the number of newborns with SCD has been increasing as well [2]. Worldwide, the prevalence of SCD is distributed unequally; the majority of cases occur in sub-Saharan Africa, India, the Middle East, and the Caribbean, but a notable number of individuals are living with SCD in North America and Western Europe as well [1, 2]. Based on these data, healthcare professionals, including obstetrics and gynecology (OB/GYN) specialists, are increasingly likely to be faced with pregnant patients with SCD, including in geographic areas where the prevalence of the disease is not considered high.

In individuals with SCD, pregnancy is associated with poorer maternal and fetal outcomes compared to the general population, including higher rates of preeclampsia and eclampsia, premature birth, increased risk of urinary tract infection, pneumonia, venous thromboembolism, and intrauterine growth retardation [3]. Furthermore, SCD-related complications like severe acute vaso-occlusive pain episodes and acute chest syndrome are more frequent during pregnancy [3].

## SCD management in pregnancy–evidence-based recommendations vs. best practice based on clinical experience

Guidelines for SCD management in adults in general, as well as during pregnancy in particular, are available [4–7]. Although these provide standardized approaches to disease management, only a small fraction of the recommendations is based on strong evidence, due to the scarcity of clinical trial data available in this population. Additionally, there are a number of aspects of pregnancy care for individuals with SCD that are not addressed at all by current professional society guidelines, leaving uncertainty about several topics, such as the role of transfusions for the prevention and management of fetal and SCD-specific maternal complications. Therefore, healthcare professionals are often left to rely on their best clinical judgement when faced with a pregnant patient with SCD.

**Fig. 1:**
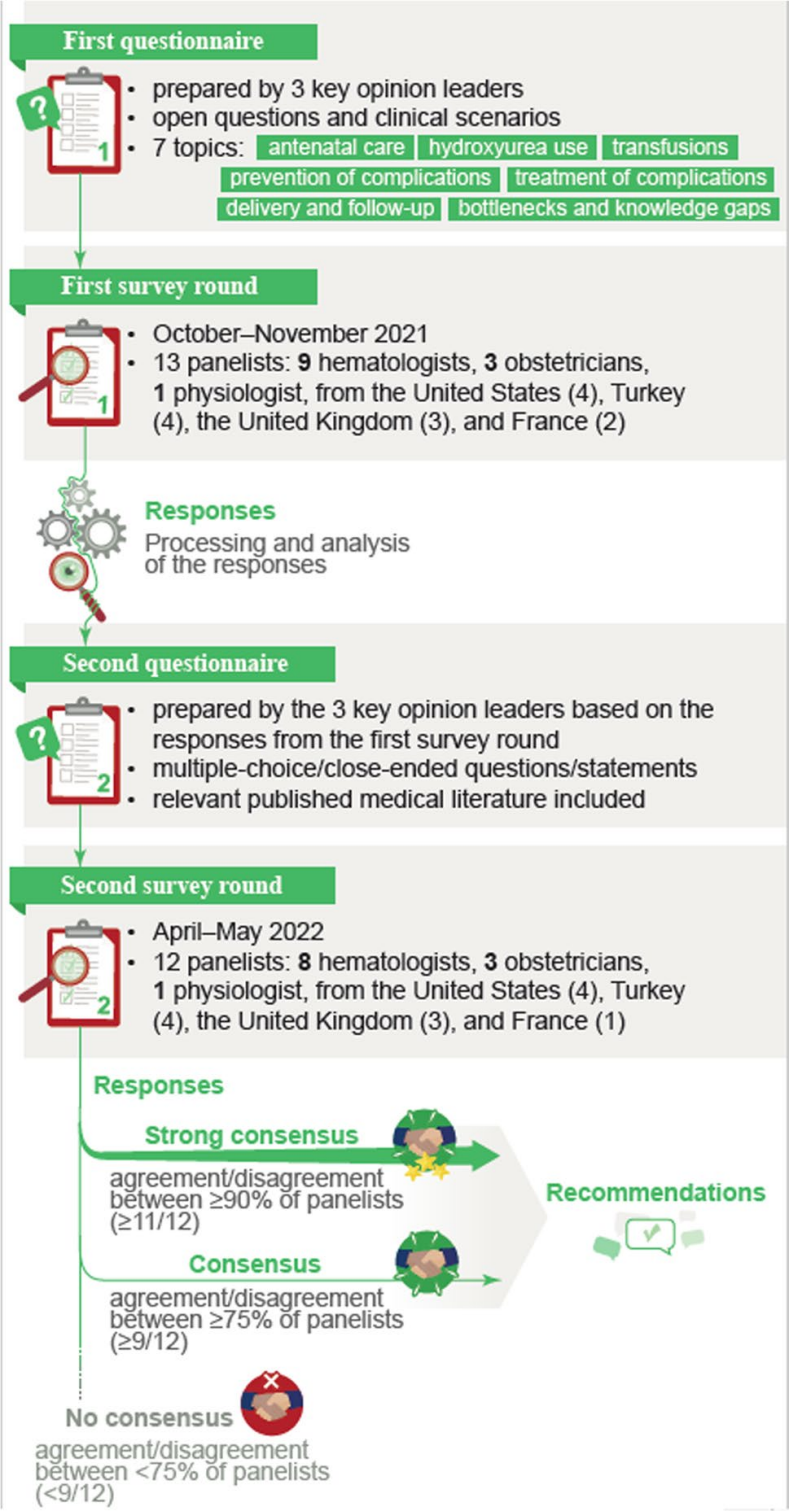
The Delphi process for obtaining consensus on topics related to SCD management in pregnancy SCD, sickle cell disease

**Fig. 2:**
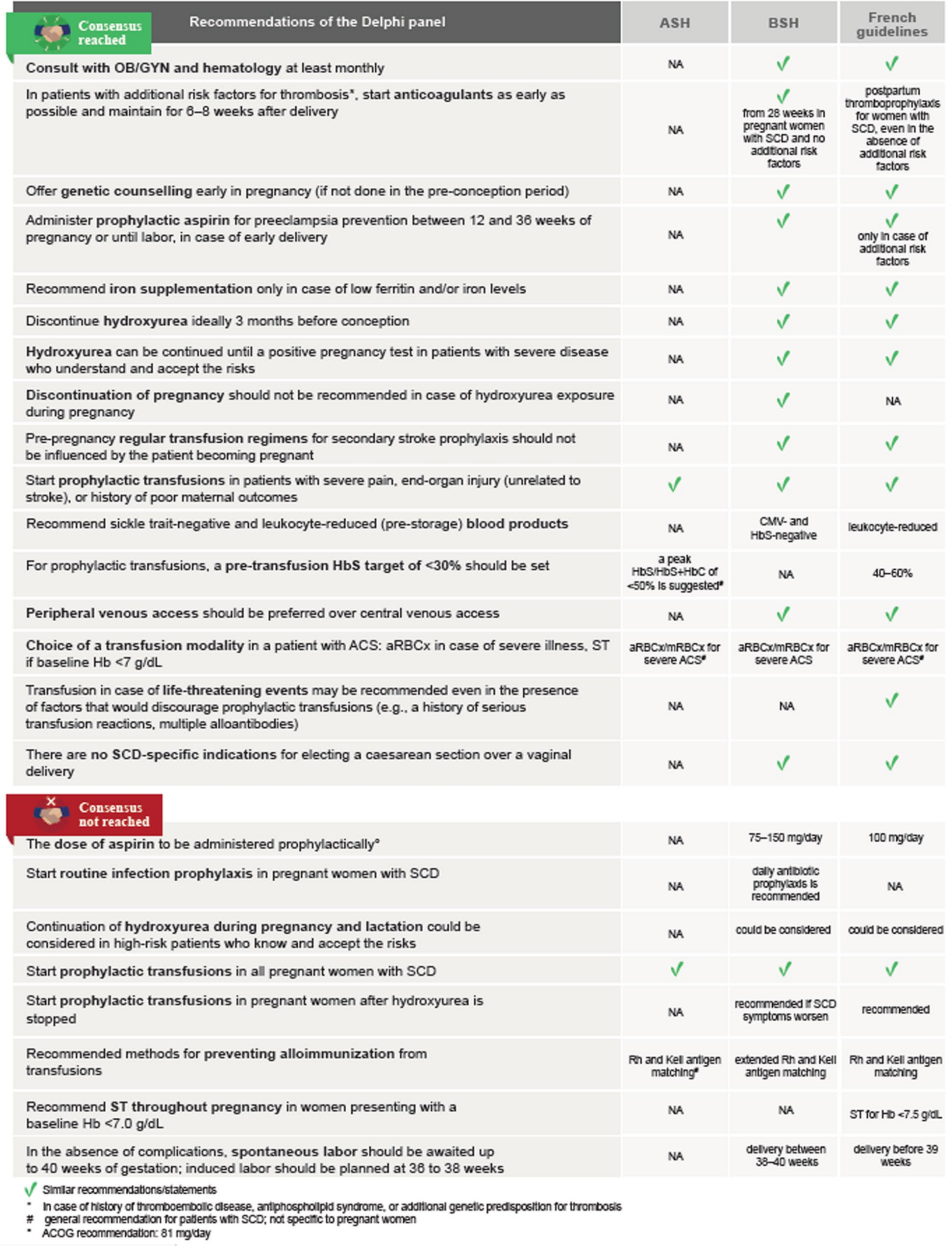
Recommendations based on consensus and their alignment with currently available guidelines on SCD management in pregnancy SCD, sickle cell disease; OB/GYN, obstetrics and gynecology; ASH, American Society of Hematology; BSH, British Society for Haematology; ACOG, American College of Obstetricians and Gynecologists; NA, not applicable; CMV, cytomegalovirus; Hb(S), (sickle) hemoglobin; HbC, hemoglobin C; ACS, acute chest syndrome; aRBCx, automated red blood cell exchange; mRBCx, manual red blood cell exchange; ST, simple transfusion

In an effort to elaborate recommendations for managing SCD during pregnancy where published evidence is limited or does not exist, we created an international, multidisciplinary Delphi panel comprised of 12 leading experts with extensive experience in SCD management (Fig. 1). After two rounds of the Delphi survey, consensus was reached in several areas of SCD management, allowing for recommendations to be formulated. A summary of these recommendations, as well as their alignment with professional societies/national guidelines, is presented in Fig. 2. The manuscript describing in detail the findings of this international Delphi panel was recently published in the journal *Blood Advances* [8]. However, we believe that the topic is of interest for OB/GYN specialists as well, who could benefit from the recommendations reported here.

## Consensus-based recommendations issued by a multidisciplinary, international Delphi panel

Areas of consensus highlighted the importance of having a multidisciplinary team providing care for pregnant patients with SCD: experts recommended at least monthly check-ups with OB/GYN and hematology specialists, and with other disciplines (e.g., pulmonology, cardiology, mental health services/psychiatry) if a clinical need arises. The experts also strongly agreed that OB/GYN specialists are the ones who should decide how often pregnant patients with SCD undergo ultrasounds and Doppler scans for fetal growth monitoring. Although strong consensus was reached in recommending a blood pressure check and urinalysis at least monthly (based on the higher risk of preeclampsia and eclampsia in pregnant individuals with SCD) [3], only eight of the 12 panelists believed that this monitoring should be performed by OB/GYN specialists. Moreover, experts strongly agreed that the decision for labor induction or elective cesarean section should be taken by a multidisciplinary team that includes OB/GYN specialists, hematologists, neonatologists, anesthesiologists, and blood bank specialists.

Another topic on which consensus was reached was the recommendation to administer prophylactic aspirin for the prevention of preeclampsia between gestational weeks 12–36, considering that this patient population has a higher risk of developing hypertensive disorders during pregnancy [3]; this is in line with recommendations of other professional societies [5–7]. However, no agreement was reached regarding the optimal dose of prophylactic aspirin, with some of the panelists indicating that they would defer this decision to OB/GYN specialists. Of note, the doses of prophylactic aspirin recommended by the various guidelines that address this topic also vary, as detailed in Fig. 2 [5–7].

In line with the currently available guidelines [5–7], the experts did not recommend starting prophylactic transfusions in all pregnant patients with SCD. However, several situations in which the experts believed prophylactic transfusion therapy would be warranted were identified, including patients with a history of poor maternal outcomes during previous pregnancies (e.g., several hospitalizations, severe SCD-specific pain), as well as the occurrence of end-organ injury unrelated to stroke during pregnancy (e.g., pulmonary hypertension, acute or chronic kidney disease). Experts recommended transfusions in cases of intrauterine growth restriction and fetal distress due to maternal complications, likely caused by uteroplacental dysfunction/insufficiency [3]. Similar to other published recommendations, experts of the Delphi panel agreed that there are no SCD-specific indications for preferring cesarean section over vaginal delivery, and that standard obstetric indications should be followed.

## Challenges of caring for pregnant patients with SCD

Currently available guidelines for SCD management in pregnancy, as well as the results of this Delphi panel comprising multiple areas for which consensus was not reached, highlight that evidence-based recommendations in this population are limited [4–8]. While recommendations based on clinical experience or expert consensus are useful for optimizing medical decision-making, attention must be drawn to the numerous knowledge gaps still present around SCD management during pregnancy that we highlighted in this Delphi panel. More rigorous studies focused on maternal and fetal outcomes such as the HELPFUL (ClinicalTrials.gov identifier: NCT04093986), PIPSICKLE (ClinicalTrials.gov identifier: NCT05253781), and TAPS-2 (ClinicalTrials.gov identifier: NCT03975894) trials are urgently needed to address these critical gaps in the field and to better inform clinical care for individuals with SCD who wish to become pregnant; however, we note that these studies were published after the Delphi panel and therefore did not inform the expert recommendations.

The multidisciplinary nature of SCD management in pregnancy is universally acknowledged. Current guidelines have mostly been issued by hematological societies and associations. Although some of the current guidelines were developed by involving OB/GYN specialists [4, 5], guidance formulated specifically for this group of healthcare professionals is scarce, and there is a paucity of established guidelines to highlight indications for utilizing interventions such as red blood cell transfusions to prevent or manage fetal complications [7]. During the Delphi process, experts identified several areas of SCD management during pregnancy where evidence is missing or incomplete, some of which are detailed in the recently published paper [8]. In addition, the lack of published conclusive evidence in several research topics was highlighted. Such topics include the safety of approved disease-modifying treatments in pregnancy, the use of steroids for fetal lung maturation, and the risk-benefit ratio of different methods of labor induction. The potential impact of prophylactic transfusion therapy during pregnancy on adverse neurodevelopment outcomes in children could also be worth exploring, considering that associations have been found between autism/neurodevelopmental disorders and several risk factors common in patients with SCD (such as anemia, hypoxia, opioid use leading to neonatal abstinence syndrome, preterm birth and intrauterine growth restriction resulting in premature/low birth weight newborns) [9].

A key strength of this work is that it reflects a diverse international expertise, which is an important aspect in the context of clinical decision-making. Ethical considerations and practical care options are strongly influenced by the setting in which they are to be delivered, with substantial differences between high- and low-resource environments. Judicious use of resources is crucial in the low-income settings where most individuals with SCD reside; nevertheless, there is a limited number of publications that address recommendations for managing SCD pregnancies in such low-resource settings [10], and the World Health Organization has convened a working group for this purpose, resulting in the recent publication of their guideline [11]. In this context, increasing the available body of evidence on the different aspects of SCD management in pregnancy is also needed to establish the cost-effectiveness of each intervention, albeit management strategies with a proven clinical benefit may also reduce serious fetal and maternal complications in ways that are beyond measure. Importantly, the applicability of clinical evidence and recommendations will inherently vary across settings, due to differences in healthcare infrastructure and resource availability. However, data from studies conducted in Africa suggest that coordinated pregnancy care models and structured clinical programs can significantly improve outcomes even in resource-limited environments [12].

## Conclusion

The publication of Sharma et al. [8], which presents the results of the first international study to use the Delphi technique to reach expert level consensus for multidisciplinary topics addressing SCD management during pregnancy, could provide essential guidance to hematology and OB/GYN specialists and organizations at large. Importantly, the Delphi panel represents not only a consensus-building effort but also a foundation for defining research still required to develop definitive, evidence-based recommendations. Advancing this field will require coordinated research efforts and international collaboration aimed at transforming outcomes for pregnant individuals with SCD.

## Data Availability

Not applicable.

## Abbreviations

SCD: sickle cell disease
HbS: sickle hemoglobin
OB/GYN: obstetrics and gynecology

## References

[1] Tebbi CK. Sickle Cell Disease, a Review. Hemato. 2022;3(2):341–66.

[2] Thomson AM, McHugh TA, Oron AP, Teply C, Lonberg N, Vilchis Tella V, et al. Global, regional, and national prevalence and mortality burden of sickle cell disease, 2000–2021: a systematic analysis from the Global Burden of Disease Study 2021. Lancet Haematol. 2023;10(8):e585–99. 37331373 10.1016/S2352-3026(23)00118-7 PMC10390339

[3] Jain D, Atmapoojya P, Colah R, Lodha P. Sickle cell disease and pregnancy. Mediterr J Hematol Infect Dis. 2019;11(1):e2019040. 31308916 10.4084/MJHID.2019.040 PMC6613624

[4] Chou ST, Alsawas M, Fasano RM, Field JJ, Hendrickson JE, Howard J, et al. American Society of Hematology 2020 guidelines for sickle cell disease: transfusion support. Blood Adv. 2020;4(2):327–55. 31985807 10.1182/bloodadvances.2019001143 PMC6988392

[5] Oteng-Ntim E, Pavord S, Howard R, Robinson S, Oakley L, Mackillop L, et al. Management of sickle cell disease in pregnancy. A British Society for Haematology guideline. Br J Haematol. 2021;194(6):980–95. 34409598 10.1111/bjh.17671

[6] Habibi A, Arlet J-B, Stankovic K, Gellen-Dautremer J, Ribeil JA, Bartolucci P, et al. Recommandations françaises de prise en charge de la drépanocytose de l’adulte: actualisation 2015 [French guidelines for the management of adult sickle cell disease: 2015 update]. Rev Med Interne. 2015;36(5 Suppl 1):S53–584. 10.1016/S0248-8663(15)60002-9 26007619

[7] The American College of Obstetricians and Gynecologists. Hemoglobinopathies in pregnancy. 2022. https://www.acog.org/clinical/clinical-guidance/practice-advisory/articles/2022/08/hemoglobinopathies-in-pregnancy. Accessed 5 Dec 2022.

[8] Sharma D, Kozanoğlu I, Ataga KI, Benachi A, Büyükkurt S, Lanzkron S, et al. Managing sickle cell disease and related complications in pregnancy: results of an international Delphi panel. Blood Adv. 2024;8(4):1018–29. 38206762 10.1182/bloodadvances.2023011301 PMC10879679

[9] Brucato M, Lance E, Lanzkron S, Wang X, Pecker LH. Developmental disorders in children born to women with sickle cell disease: A report from the Boston Birth Cohort. EJHaem. 2022;3(3):894–8. 36051016 10.1002/jha2.478 PMC9421989

[10] Afolabi BB, Babah OA, Adeyemo TA. Evidence-based obstetric management of women with sickle cell disease in low-income countries. Hematology. 2022;2022(1):414–20. 36485120 10.1182/hematology.2022000377 PMC9821549

[11] World Health Organization. WHO recommendations on the management of sickle-cell disease during pregnancy, childbirth and the interpregnancy period. 2025. https://www.who.int/publications/i/item/9789240109124. Accessed 12 June 2026. 40627713

[12] Asare EV, Olayemi E, Boafor T, Dei-Adomakoh Y, Mensah E, Ghansah H, et al. Implementation of multidisciplinary care reduces maternal mortality in women with sickle cell disease living in low-resource setting. Am J Hematol. 2017;92(9):872–8. 28512745 10.1002/ajh.24790 PMC7725481

